# Interindividual vocal tract diversity influences the phonetic diversification of spoken languages

**DOI:** 10.1371/journal.pbio.3004003

**Published:** 2026-10-13

**Authors:** Steven Moran, Tiena Danner, Marcia S. Ponce de León, Christoph P. E. Zollikofer

**Affiliations:** 1 Institute of Biology, University of Neuchâtel, Neuchâtel, Switzerland; 2 Linguistic Research Infrastructure, University of Zurich, Zurich, Switzerland; 3 Department of Informatics, University of Zurich, Zurich, Switzerland; 4 Center for Climate Physics, Institute for Basic Science, Busan, Republic of Korea; University of St Andrews, UNITED KINGDOM OF GREAT BRITAIN AND NORTHERN IRELAND

## Abstract

Human vocal tracts, like other physical traits, exhibit extensive interindividual variation. However, little is known about how this variation translates into phonetic diversity between speakers, and ultimately, across languages. Here we develop a biocultural model of phonetic evolution, analogous to Russell Lande’s model of multivariate phenotypic evolution. We show that individual vocal tract morphologies and associated articulatory strategies leave consistent acoustic signatures, resulting in a characteristic pattern of phonetic variation within speaker groups that correlates with the pattern observed between groups. Recalling a central tenet of evolutionary biology—that within-group variation feeds between-group diversification—our findings suggest that phonologically similar vowel systems can attain phonetically diverse realizations according to a neutral-like evolutionary process. Our model serves as a null hypothesis for disentangling the biological versus cultural mechanisms underlying phonetic diversification, where deviations from the neutral expectation may indicate culturally mediated language change.

## Abstract 2

Human vocal tracts vary extensively between individuals, but how does this translate into phonetic diversity between speakers and languages? This study uses vocal tract imaging to develop a biocultural model of phonetic evolution, helping to disentangle biological versus cultural mechanisms of phonetic diversification.

## Introduction

There is extensive interindividual variation in terms of the size and shape of the hard and soft tissues involved in speech production [1–4]. Both “nature” (natural selection, genetic drift) [5–10] and “nurture” (developmental and sociocultural factors) [11–18] shape an individual’s vocal tract morphology and imbue their voice with acoustic markers (age, gender, sex, size) [19,20] and speaker-specific voice qualities [21,22], making them sound unique [23].

Despite considerable interindividual variation, however, speakers within a speech community reach acoustically similar targets, which they perceive as the same contrastive speech sounds, i.e., phonemes [24,25]. While speaker groups thus tend to conserve phonetic (as well as other linguistic) properties, it is well known that language properties are constantly changing through time [26,27], and a key question to answer is how biology, environment, and culture together bring about these changes.

Here we focus on the biological side of the question, asking specifically how vocal tract variation within a speaker group influences phonetic variation, and how phonetic variation within speaker groups correlates with variation between groups. To the best of our knowledge, these interlinked questions have not yet been addressed comprehensively and empirically. While listener- or classification-centered studies often account for acoustic differences between speakers through normalization procedures [28–32], here we are specifically interested in the patterns of variation produced by those interindividual differences. Previous studies have focused on population-level differences in vocal tract shape [18,23,33], on specific areas of the vocal tract [3,33], or on differences related to sex, age and size [12,19,34]; others have used modeling and simulation-based approaches [2,33,35–37], or are preliminary [38–41].

In this study, we quantify the origins and propagation of interindividual variation across the successive levels of speech production—from variation in vocal tract shape and articulatory gestures to variation in the associated phonetic output within a speaker group, and from group-level to language-level phonetic variation. We use the five cardinal vowels (i, e, a, o, u) as a test case, because they are well characterized in terms of articulation and acoustics, and are shared by a wide variety of typologically diverse languages.

To quantify articulatory-acoustic relationships, we propose a new approach, *morpho-phonetics*, that combines geometric morphometric analysis [42] of the shape of the vocal tract in real-time Magnetic Resonance Imaging (rtMRI) [43] with spectral analysis of the concurrent audio signal during speech articulation (Figs 1 and S1). We examined the morpho-phonetic covariation of the cardinal vowels as pronounced by 32 speakers (16 women and 16 men) of American English from different ethnic and linguistic backgrounds. Vowels were extracted from the carrier words (‘beat’, ‘bait’, ‘pot’, ‘boat’, ‘boot’) from the USC 75-Speaker Speech MRI Database [43].

**Fig 1:**
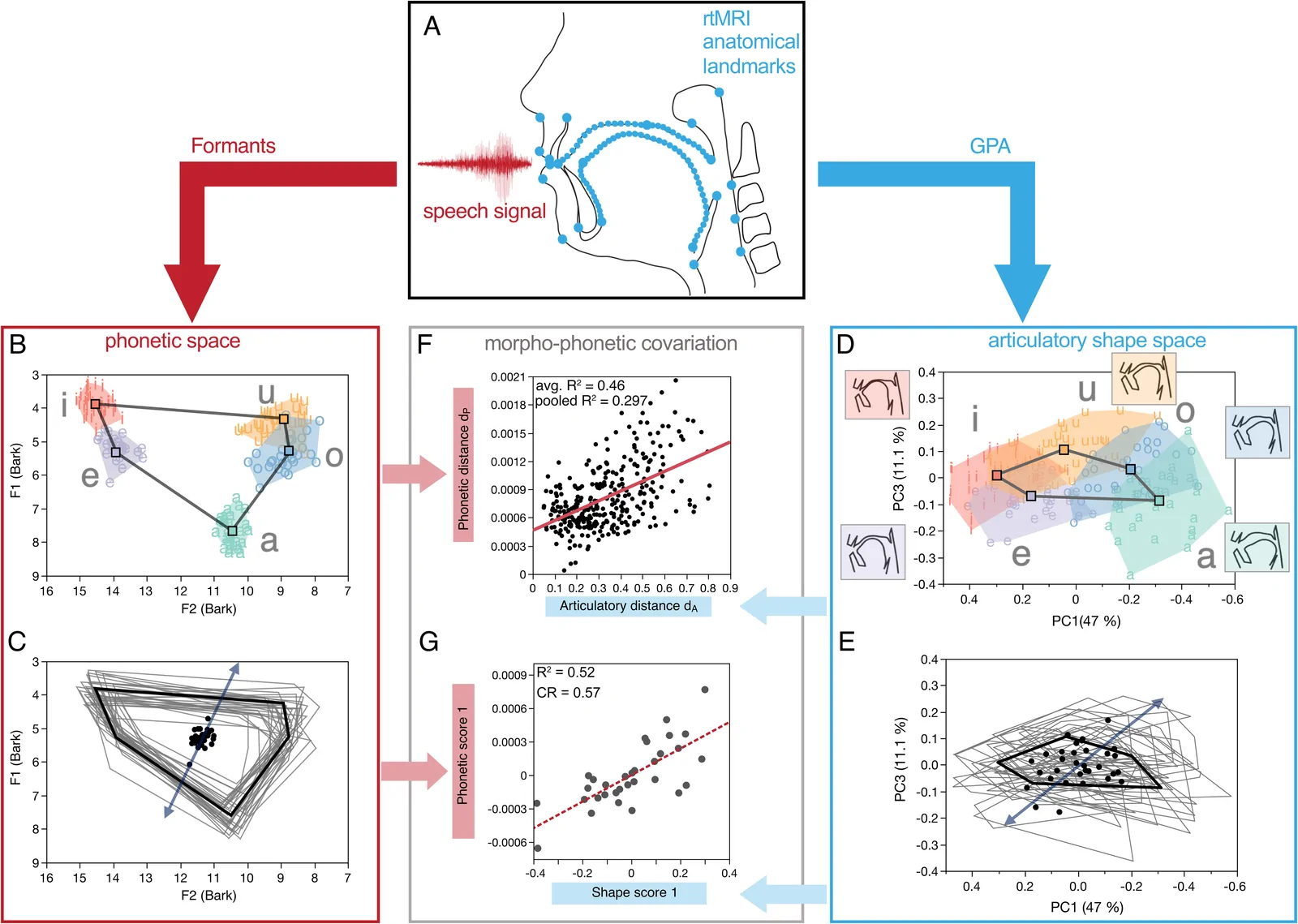
Morpho-phonetic co-analysis of vowel production. **(A)** Anatomical landmarks (blue dots) are placed on midsagittal MRI images of the vocal tract recorded during speech production. At each corresponding timestamp, acoustic analysis (formant frequency extraction) is undertaken on denoised audio recordings. **(B–C)** Resulting acoustic-phonetic variation in formant space rendered as interindividual variation of vowels (B) and vowel polygons (C). Centroids of individual acoustic vowel polygons (black dots; C) serve as proxies for acoustic timbre. **(D–E)** Corresponding variation in articulatory shape space (given by principal components PC1 and PC3) rendered as interindividual variation of vowels (D; inset graphs represent the mean shape of the vocal tract for each vowel) and vowel polygons (E). Centroids of individual articulatory vowel polygons (black dots) serve as proxies for the resting-state vocal tract morphologies. In (D) and (B), note the reduction of within-vowel variation relative to between-vowel variation in phonetic space compared to articulatory shape space, which reflects articulatory adaptation to target vowels. **(F–G)** Morpho-phonetic covariation. (F) Linear regressions of phonetic vs. articulatory distances between vowels (individual regressions with average *R*^*2*^ = 0.46; pooled regression with *R*^*2*^ = 0.297 (red line)). (G) Partial least-squares (PLS) analysis of the individuals’ phonetic vs. articulatory vowel polygon data, reflecting residual morpho-phonetic covariation not compensated for by articulatory adaptation. Details in main text and Materials and methods. The data underlying this Figure can be found at https://osf.io/aeg7p/.

For each vowel spoken by each individual, the articulatory vocal tract shape and its corresponding phonetic output were sampled at synchronous timestamps. Physically, vowel-specific articulation is achieved by coordinated jaw, lip, tongue and velum movements, whose interplay determines the shape of the vocal tract and its acoustic output. Here, vocal tract shape is quantified with a set of anatomical landmarks (*K* = 95) (Figs 1A and S2, S1 Table) placed on each rtMRI frame, and shape variation is analyzed and visualized with methods of geometric morphometrics (Fig 1A and 1D–1E). Articulation results in an acoustic signal (Fig 1A–1C), which is quantified here with vocal tract resonance frequencies, i.e., formants F1–F4, and the fundamental frequency, F0.

Geometric morphometric analysis of the vocal tract and formant analysis yield multidimensional data sets. We use principal components analysis (PCA) as a dimension reduction technique to explore the data structures and to characterize the principal patterns of variation in the data. Covariation between articulatory gestures and phonetic output (i.e., morpho-phonetic covariation) is examined with partial least-squares analysis (PLS). We focus on those patterns of variation that are shared by all individuals, independent of sex, age, and vocal tract size. The workflow and the main results of these analyses are visualized in Fig 1; details are provided in Materials and methods.

## Results

### Articulatory and acoustic vowel spaces are similar

Fig 1B shows the acoustic variation given by the first two formants (F1 and F2) on the Bark scale [44]. The five vowels are represented by separated data clusters and their centroids form together the well-known acoustic vowel polygon. Fig 1D shows the associated articulatory variation along principal components PC1 and PC3 of shape space (58.1% of total variation). PC2 accounts for interindividual differences unrelated to vowel articulation (13.3% of total variation; see S3A Fig). The articulatory shape space shows partially overlapping vowel data clusters, but notably the centroids of the clusters also form a vowel polygon, which has a similar geometric arrangement to the acoustic vowel polygon. The articulatory and acoustic vowel polygons therefore have corresponding shapes. To quantify the correspondence between articulatory and acoustic vowel polygons, we studied how an individual’s articulatory discrimination between vowels translates into acoustic discrimination. To do so, we computed for each individual all 10 between-vowel distances in both articulatory and acoustic spaces. Fig 1F shows that articulatory distances correlate with acoustic distances (average of individual speaker correlations: *R*^*2*^ = 0.46; pooled data: *R*^*2*^ = 0.297).

### Articulatory gestures are adapted to speaker-specific vocal tract morphology

The moderate correlation between articulatory and acoustic contrasts (Fig 1F) reflects the fact that the individuals in our sample have widely different vocal tract morphologies, and they use a remarkably wide range of articulatory gestures to produce any given target vowel (see S4–S8 Figs). Therefore, we investigated how individual vocal tract morphology influences speakers’ articulatory gestures, and how these gestures translate into acoustics. A comparison of Fig 1B and 1D shows that there is substantially more articulatory than acoustic variation around corresponding vowel centroids. To quantify this effect, we evaluated the ratio of within-vowel to between-vowel variance (variance ratio; VR). We found that VR is consistently larger in articulatory space (VR_articulatory_ = 0.548) than in acoustic space (VR_formant_ = 0.065; see also S2 Table). Thus, a wide range of individual articulatory gestures results in a narrower range of acoustic variation, supporting earlier evidence that individuals adapt their articulatory gestures to produce similar-sounding vowels [45,46]. To further explore the inner workings of articulatory adaptation, we examined how an individual’s palate shape influences the shape of their tongue during vowel production (Fig 2). To do so, we calculated the mean shape of each individual’s palate and tongue, averaged over all vowels. PLS analysis shows a significant influence of palate shape on tongue shape (Fig 2A, see Materials and methods). Interindividual differences in palate shape therefore translate into systematic differences in articulatory tongue shape. Specifically, individuals with short, rounded hard palates produce more rounded tongue gestures than individuals with elongated and shallow palates (Fig 2B). Fig 2C indicates that articulatory adaptation of the tongue tends to maintain a standard cross-sectional air tube profile in the anterior region of the oral cavity, but less so in the posterior region. Overall, differences in vocal tract morphology are only in part acoustically compensated through articulatory adaptation.

**Fig 2:**
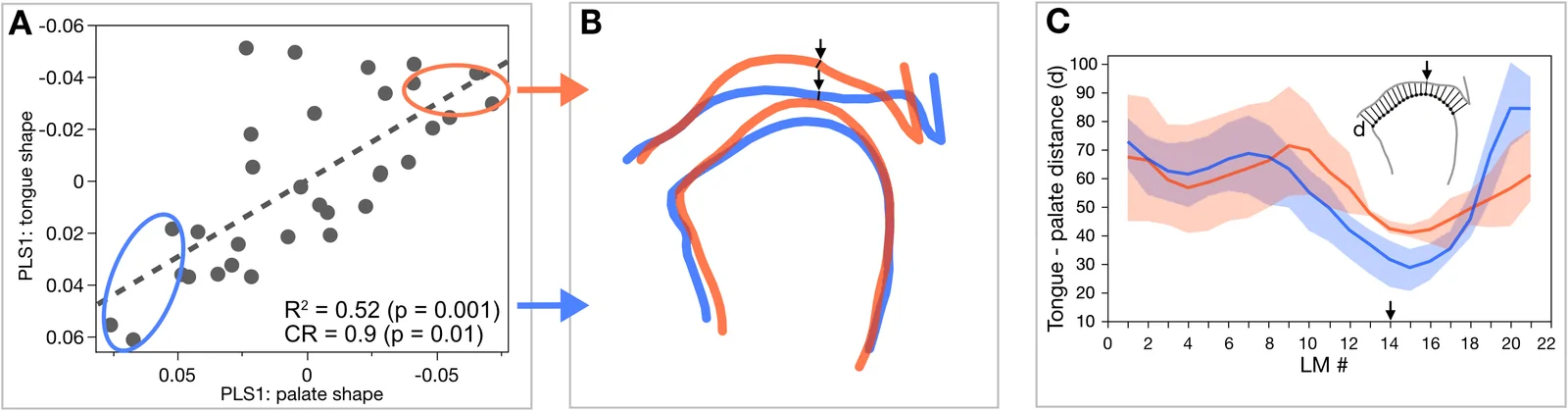
Articulatory adaptation of the tongue to individual palate shape. **(A)** Covariation between tongue and palate shapes is computed with partial least-squares analysis (PLS). The first pair of PLS components (PLS1) comprises 67.5% of the total covariation. **(B)** Articulatory adaptation is visualized for the opposite ends of the morphological variation in (A) (average morphologies of the three individuals enclosed in red and blue circles, respectively); black arrows indicate the border between the hard and soft palate. Note that in a short/rounded palate (red) the dorsum of the tongue assumes a more rounded shape. **(C)** Air tube cross-sectional profiles derived from the morphologies in (B) (red/blue lines and bands represent group-specific averages and ranges). LM # indicates landmark position along tongue dorsum; (d) denotes tongue-palate distance shown in the inset graph. The data underlying this Figure can be found at https://osf.io/aeg7p/.

### Individual vocal tract morphology results in acoustic differences between speakers

Next we asked how the residual, uncompensated acoustic differences between individuals are related to the underlying differences in their vocal tract morphologies. To do so, we investigated the covariation between individual vowel polygon geometries in articulatory and formant space (Fig 1G). A lack of covariation would indicate that the acoustic properties of the target vowels are independent of their individual articulatory properties, indicating full compensation for individual vocal tract differences. However, PLS analysis shows significant covariation (Fig 3A, see Materials and methods), confirming that articulatory adaptation is only partial (Fig 2B and 2C), such that individual differences in the vocal tract (Fig 3A) lead to systematic acoustic differences between speakers (Fig 3B). This effect is visualized by the real-life morphological and acoustic properties of individuals at opposing ends of the data scatter in Fig 3B and 3C: those with rounded/short versus shallow/elongated palates. Specifically, differences in palate shape result in different timbre of the voice (as indicated by shifts in the position of the vowel polygon in acoustic space), different acoustic range (change in the size of the polygon), and different acoustic contrasts between vowels (change in the shape of the vowel polygon; Fig 3B and 3C). In sum, our analyses show that interindividual variation in vocal tract morphology generates correlated variation in vowel acoustics.

**Fig 3:**
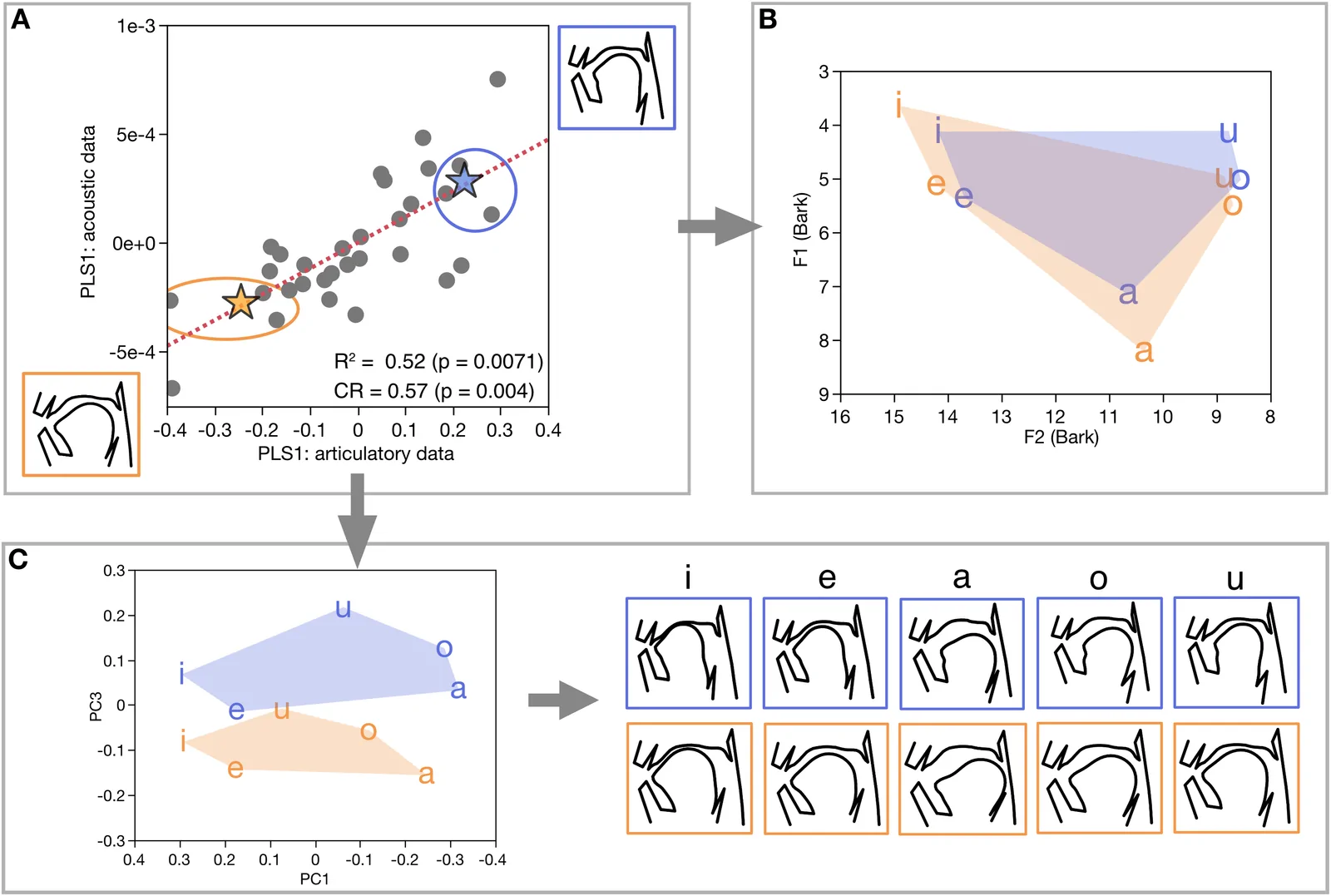
Patterns of uncompensated morpho-phonetic covariation. **(A)** PLS analysis of the covariation between articulatory and acoustic vowel polygons (same data as in Fig 1G). The first pair of PLS components (PLS1) comprises 49.8% of the total covariation. Each data point represents joint articulatory and acoustic data of one individual. Orange/blue stars represent the mean of individuals at the opposite ends of the data scatter (individuals in orange/blue circles) and are used for the visualization of contrasting articulatory morphologies (C) and corresponding acoustics (B). **(B)** Corresponding acoustic vowel polygons in F1–F2 formant space (Bark scale). **(C)** Articulatory vowel polygons and associated articulatory differences between individuals with rounded/short and shallow/elongate palates. The data underlying this Figure can be found at https://osf.io/aeg7p/.

### Vocal tract variation influences within- and between-language phonetic diversity

Fig 3 has shown for our morpho-phonetic sample of English speakers (MP-E) that vocal tract variation systematically conditions vowel variation. Yet the more general question remains: how do vocal tract variation and the ensuing acoustic variation between speakers ultimately influence phonetic diversification across languages? Given the current lack of cross-linguistic morpho-phonetic data, we addressed this question by combining the MP-E data with the multilingual speech data in the VoxCommunis Corpus (VC-C) [47]. We analyzed phonetic variation of the cardinal vowels in 10 typologically diverse languages of the VC-C sample (Basque, Bulgarian, Hindi, Italian, Portuguese, Romanian, Russian, Tamil, Turkish, Vietnamese) and compared it with the corresponding variation in the MP-E sample. First, we pooled the data of the 10 VC-C languages to evaluate within-language variation in the sample (see Materials and methods). Fig 4 shows that the pattern of within-language variation in the VC-C sample (gray ellipses) is similar to that in the MP-E sample (dashed gray ellipses) (multivariate comparison of variances: *p* = 0.63; see Materials and methods). Since the MP-E phonetic variation is conditioned by vocal tract variation (Fig 3A), we infer that there exists a basic pattern of speaker-level phonetic variation within languages that is grounded, at least in part, in the constraints imposed by vocal tract variation among speakers. Second, we used the centroids from the 10 languages to evaluate the pattern of between-language variation in the VC-C sample (Fig 4, red ellipses). Together, these analyses show that variation between languages is similar to variation within languages (red and gray ellipses in Fig 4, respectively) (multivariate comparison of variances: *p* = 0.33), suggesting that similar processes govern the phonetic diversification within and between languages.

**Fig 4:**
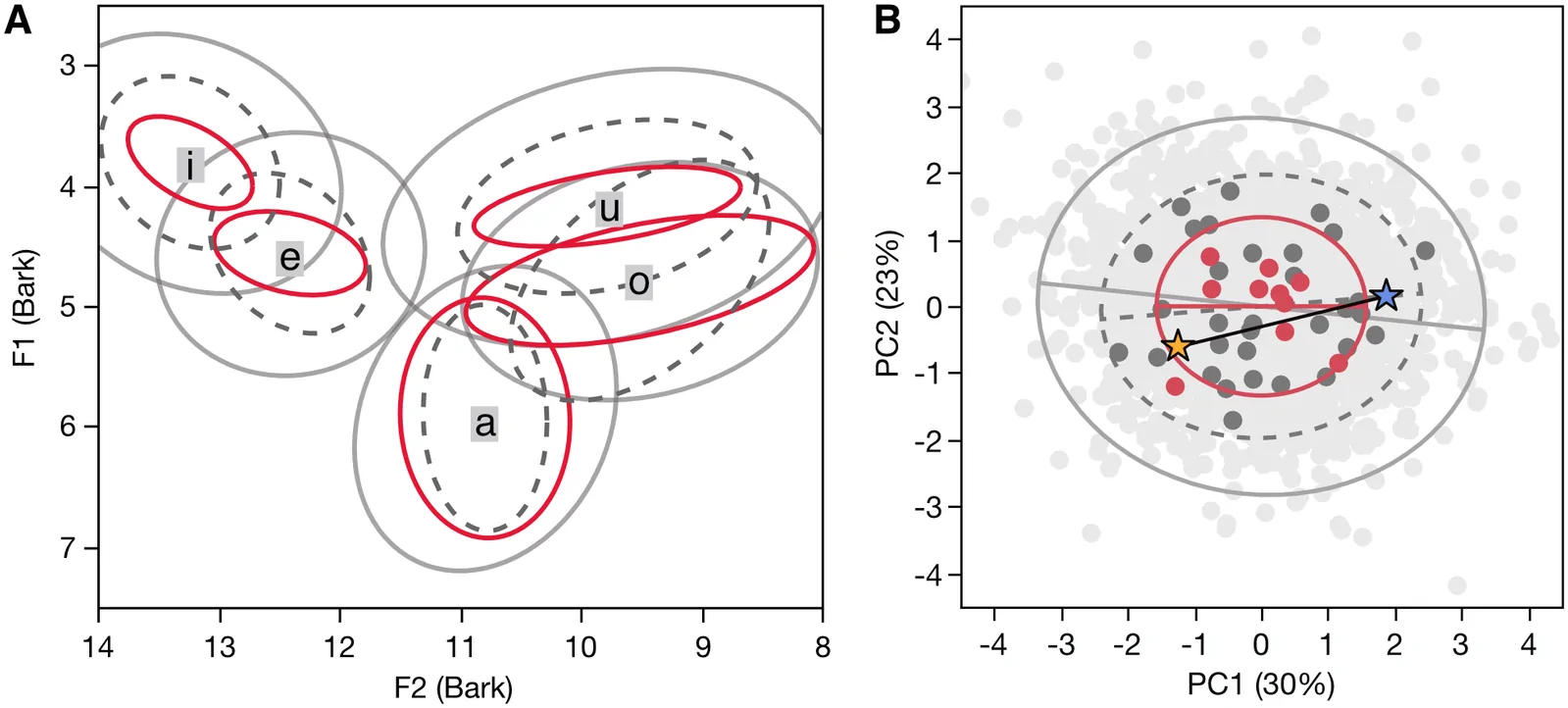
Patterns of within-language and between-language variation in cross-linguistic speech acoustics data. **(A)** Acoustic variation of the cardinal vowels in F1–F2 formant space. Ninety percent density ellipses show variation for the morpho-phonetic English speaker data (MP-E) (dark gray dashed lines), the pooled within-language data from the VoxCommunis Corpus (VC-C) (light gray solid lines) and the between-language VC-C data (red). Note the similarity in direction of variation across the different groups, which indicates a shared pattern of variation across the different data subsets. **(B)** PCA of vowel polygon variation (90% density ellipses with major axes; colors as in (A)). Gray dots represent individuals (light gray: VC-C, dark gray: MP-E); red dots represent VC-C language means. The line connecting the yellow and blue stars marks phonetic variation due to vocal tract variation (MP-E data from Fig 3A). The data underlying this Figure can be found at https://osf.io/aeg7p/.

## Discussion

Previous studies have shown that speech sounds convey significant information about an individual’s physical characteristics (such as body size and sex), social status, and emotional state [22,48], resulting in considerable inter-speaker phonetic variability within a speaker group. Precisely because of its ubiquity, inter-speaker phonetic variability is often accounted for as “noise” that tends to blur the underlying textual and/or structural speech signal. For example, during categorization tasks such as automatic speech recognition [30], individual acoustic features are typically filtered out to focus on the “pure” speech signal. Likewise, in studies investigating language diversification with methods of phylogenetic inference, the “phenotype” of each language is characterized by structural linguistic features that are largely independent of the individual properties of the actual speakers [49–51].

As a complement to such top-down approaches, we have taken a bottom-up approach here that starts with a focus on individual morpho-phonetic features. While these features are statistically independent of sex and vocal tract size (see Materials and methods), we have shown that they carry significant information about the shape of an individual’s vocal tract. We argue here that the underlying pattern of interindividual vocal tract variation is not only a source of phonetic variation within speaker groups, but also a potential source of phonetic variation and diversification between groups. To formalize this idea, we propose a biocultural model of phonetic diversification, by analogy to Lande’s well-established biological model of multivariate phenotypic evolution [52].

In Lande’s model, the multivariate phenotypic variability ${𝐕}_{p}$ in a population is the sum of the multivariate genotypic variability ${𝐕}_{g}$ and external influences ${𝐕}_{e}$ (environment, other factors, stochasticity):


$$\begin{array}{c} {𝐕}_{p}={𝐕}_{g}+{𝐕}_{e}. \end{array}$$ (1a)

${𝐕}_{p}$ and ${𝐕}_{g}$ are variance-covariance matrices that quantify internal phenotypic constraints (${𝐕}_{p}$: covariation between phenotypic traits) and genotypic constraints (${𝐕}_{g}$: covariation between genotypic traits), respectively.

Lande’s model further states that change Δ𝐳 in the phenotype ${𝐕}_{p}$ over a given time span (e.g., one generation) can be expressed as:


$$\begin{array}{c} \Delta 𝐳={𝐕}_{p}\beta +\epsilon , \end{array}$$ (2a)

where β is a selection-gradient vector and ϵ is a stochastic term. Under neutral evolution (β=0), there is no directional change in ${𝐕}_{p}$. However, stochastic effects ϵ (random mutations, generational “subsampling” from finite-sized populations) result in genetic drift. As a result, the variation between subpopulations ${𝐕}_{p.b}$ evolves as a function of the variation within populations ${𝐕}_{p.w}$:


$$\begin{array}{c} {𝐕}_{p.b}={𝐕}_{p.w}\frac{t}{N_{e}}, \end{array}$$ (3a)

where *t* is time in generations and $N_{e}$ is the effective population size. This expresses a central tenet of evolutionary biology: within-group variation feeds processes of between-group diversification.

While the Lande model considers how genotypic variance in a population generates phenotypic variance, our biocultural model of phonetic diversification considers how, in a speaker group, phenotypic variation ${𝐕}_{v}$ of the vocal tract (both morphological and articulatory) generates acoustic variation ${𝐕}_{a}$:


$$\begin{array}{c} {𝐕}_{a}={𝐕}_{v}+{𝐕}_{e^{\prime }}, \end{array}$$ (1b)

where ${𝐕}_{e^{\prime }}$ stands for external influences and stochasticity. In general terms, ${𝐕}_{v}$ and ${𝐕}_{a}$ represent multivariate vocal tract and acoustic phenotypes, respectively, rather than individual anatomical or phonetic features.

In the present study, we examine specific empirical subsets of these general quantities: ${𝐕}_{v}$ is represented by midsagittal two-dimensional vocal tract landmarks, while ${𝐕}_{a}$ is represented by the five-vowel constellation in formant space. ${𝐕}_{v}$ captures the articulatory covariation among the five vowels, while ${𝐕}_{a}$ captures the acoustic covariation. These variance-covariance matrices thus quantify the effects of past and current constraints imposed by the shapes of the speakers’ vocal tracts and articulatory movements—as well as the constraints imposed by the listeners’ requirement for vowel distinguishability. The empirical scope of the model can be expanded by representing ${𝐕}_{v}$ with three-dimensional vocal tract morphology and time-resolved articulatory variation, and by broadening ${𝐕}_{a}$ to include additional speech sounds (such as consonants) and further acoustic dimensions (such as MFCCs), e.g., [53]. Both quantities could also be estimated in more diverse morpho-phonetic samples.

Following Lande’s logic [52], the expected change in a speaker group’s five-vowel constellation over time can be expressed as follows:


$$\begin{array}{c} \Delta 𝐳={𝐕}_{a}\beta +\epsilon^{\prime }, \end{array}$$ (2b)

where Δ𝐳 denotes change in the multivariate vowel constellation and β represents a selection-gradient vector, and $\epsilon^{\prime }$ is a stochastic term. This vector is broadly construed as the perceptual, social, or cultural constraints and biases that act on acoustic variation—both currently and in the future. In this model, acoustic variation is constantly injected into the group’s speech system through vocal tract variation ${𝐕}_{v}$ in Eq (1b), and is removed from the system through vowel distinguishability selection β.

Under a neutral evolutionary scenario (β=0), speaker groups tend to drift apart stochastically, such that the variation between groups ${𝐕}_{a.b}$ evolves as:


$$\begin{array}{c} {𝐕}_{a.b}={𝐕}_{a.w}\frac{t}{N_{e}}, \end{array}$$ (3b)

where ${𝐕}_{a.w}$ is the variation within speaker groups, *t* is time (in generations), and $N_{e}$ is the effec*t*ive group size (all individuals involved in communication). According to this neutral model, the between-language and within-language components of the acoustic phenotype are expected to remain proportional to each other, yielding a slope close to one in the log–log variance regression [52,54,55] (S9 Fig). Note that the first principal component of ${𝐕}_{a}$ (Fig 4B) accounts for the largest proportion of acoustic variance and, in our model, represents the direction of greatest vowel polygon “evolvability”.

Overall, model Eqs (1b)–(3b) formalize our hypothesis that vocal tract and articulatory variation within speaker groups influences phonetic variation, and that within-group patterns of variation are mirrored in patterns of phonetic diversification between groups.

We can now assess to what extent our results fit the neutral expectations of our biocultural model. In our MP-E sample, we found that articulatory gestures and the corresponding acoustics have similar vowel polygon representations in morphometric and phonetic spaces, respectively. While articulatory vowel polygons have not yet been reported, broadly similar vowel polygon representations have been found in neuromotor and neurosensory analyses [56–65]. Further studies are needed to elucidate the complete sequence of vowel representations, from neuromotor pattern generation to articulatory movements, acoustic output and speech perception [66–68].

We have also shown that the morphology of an individual’s vocal tract leaves a consistent signature on their articulatory gestures and the associated phonetic output. Speakers compensate for anatomical differences when producing language-specific vowel targets, but this compensation is incomplete, leaving a measurable morpho-phonetic residual. Consequently, our MP-E data show that acoustic variation is influenced by vocal tract variation among speakers (Figs 2 and 3). Our PLS analysis indicates that ${𝐕}_{v}$ accounts for about 50% of the total variation in ${𝐕}_{a}$, while ${𝐕}_{e^{\prime }}$ comprises the remaining variation. The topological similarity of the vowel polygons in articulatory and acoustic spaces indicates that the linear model Eq (1b) is a fair approximation of morpho-phonetic covariation. Figs 1 and 4 show that the between-vowel acoustic covariation is strongly structured, with minimal overlap between neighboring vowels, likely reflecting the distinguishability constraints mentioned above.

We further found that the VC-C language sample studied here exhibits patterns of within-group acoustic variation similar to that in the MP-E sample (Fig 4). We hypothesize that this basic pattern of within-group acoustic variation reflects, at least in part, the same underlying vocal tract, articulatory and distinguishability constraints observed in the MP-E data. Currently, this hypothesis cannot be tested formally, because the VoxCommunis data sets are acoustic only, such that morpho-phonetic covariation cannot be established.

Finally, our data show that variation in vowels across languages is proportional to variation within languages. This pattern is consistent with the predictions of Eq (3b). This proportionality suggests that the variation of the vowel polygon between languages exhibits the same structured variation that exists among speakers within groups (Fig 4A). This is reflected by the slope, *b*, which is approximately equal to 1 in the log–log plot (S9 Fig). Non-neutral change, by contrast, is suggested when perceptual, social, cultural, environmental, or historical biases act differentially on particular axes of vowel polygon variation, thereby producing deviations from the expected slope of one [54,55]. However, a slope of b≅1 is also compatible with a “neutral-like” evolutionary scenario under weak stabilizing selection [69]. In our case, this would imply that the amount of acoustic vowel polygon variation introduced through vocal tract variation (morpho-phonetic covariation) is approximately equal to the amount of variation removed through stabilizing selection (vowel distinguishability).

It is often assumed that the biological “language evolution” was completed with the acquisition of the language faculty, and that all subsequent “language change” is due to cultural transmission and historical change [70]. This view has recently been challenged on both methodological and empirical grounds, indicating that biological processes still influence the course of spoken language evolution [18,33,71]. This study contributes to the existing body of evidence by demonstrating that vocal tract variation influences phonetic diversity among speakers, and that phonetic diversity within languages influences diversity between languages. Future studies should expand the limited morpho-phonetic sample examined here to include a wider range of languages and phonemes. The “neutral-like” biocultural model of phonetic diversification can then serve as a null hypothesis to investigate patterns of directional and diversifying selection in spoken language evolution. Additionally, these results should be compared with those obtained on other species, particularly vocal learners (cf. [72]).

## Materials and methods

### Morpho-phonetic data of English speakers (MP-E)

We used a subset of the USC 75-Speaker Speech MRI Database [43], an open-source database consisting of video and concurrent audio recordings of linguistically motivated speech tasks acquired during real-time Magnetic Resonance Imaging (rtMRI) of the upper vocal tract. The rtMRI data were acquired at 83.28 frames per second at an image matrix size of 84×84 pixels. Synchronous audio recordings were obtained in the scanner using fiber-optic microphones and were then denoised and are available in .wav format.

We extracted carrier words (‘beat’, ‘bait’, ‘pot’, ‘boat’, ‘boot’) to analyze American English vowels (i, e, a, o, u) as produced (each twice) by *N* = 32 speakers (16 females and 16 males; aged 18–59 y) from different ethnic and linguistic backgrounds. For each speaker, MRI frames were extracted from the rtMRI videos (see S1 Fig). Depending on the length of the vowel, around 10 consecutive images (corresponding to 0.12 s) were extracted. In total, 3,210 rtMRI images were used for the morphometric analyses (32 speakers, ∼100 images each across two repetitions of five vowels). The audio data were synchronized with the extracted image positions for acoustic-phonetic analysis of the vowels. Thus, each extracted set of images and synchronous timestamps in the audio recordings together represent a vowel visually and acoustically.

### Geometric morphometric analysis of vocal tract shape

The rtMRI images were resampled to 164×164 pixels for geometric morphometric (GM) analysis [42,73]. The shape of the upper vocal tract was quantified with *K* = 95 two-dimensional landmarks, denoting both stationary skeletal and soft-tissue structures (philtrum, anterior nasal spine, upper incisor tip, hard palate, inferior end of the nasopharynx, anterior borders of intervertebral disks spanning vertebrae C2 to C5) and moving skeletal and soft-tissue structures (lips, chin, supramentale, tongue, soft palate, uvula, epiglottis). The landmarks consisted of anatomical reference points (*K*_*f*_ = 21) and semilandmarks (points along curves between reference points; *K*_*c*_ = 74). See S2 Fig and S1 Table.

Landmark positions were digitized semi-automatically on each of the 3,210 rtMRI images using the R software packages *StereoMorph*, *Morpho* and *geomorph* [74–77]. Generalized Procrustes Analysis (GPA) [73] was carried out to scale individual landmark configurations to unit centroid size and to minimize differences due to rotation and translation of the individual configurations. GPA was performed with only those hard-tissue landmarks that remain stationary during articulation (see S2 Fig and S1 Table). For each vowel of each speaker, repeated rtMRI frames and the two recorded productions were averaged to obtain one mean articulatory configuration per vowel and speaker. The morphometric data matrix used for all further analyses thus consisted of 5*N* = 160 rows (representing the 5 vowels per *N* = 32 individuals) and 2*K* = 190 columns (representing the 2D Procrustes shape coordinates of the *K* = 95 landmarks per configuration). Principal Components Analysis (PCA) was then performed on the Procrustes shape coordinates to create a low-dimensional representation of shape space. One major advantage of GM analysis is that each articulatory shape of the vocal tract (per individual and per vowel) is represented by one multidimensional point in shape space. Conversely, each point in shape space can be re-transformed into its corresponding vocal tract shape in 2D physical space. This property facilitates the identification and visualization of major patterns of shape variation in the sample (see Figs 1–3).

### Acoustic data extraction

Vocal tract resonance frequencies (formants F1–F4) and the fundamental frequency (F0) were extracted from the audio recordings of the rtMRI videos at synchronous timestamps with the image frames. Vowel measurements were taken through visual inspection of the spectrograms using Praat [78]. This procedure is common practice given the unreliability of automatic formant trackers [79], particularly in noisy environments (e.g., MRI scanner noise is present in higher frequency ranges). To assess measurement reliability, a random subset of 50 tokens was remeasured by the same observer, who was blinded to the original values. This subset comprised the five target vowels from 10 speakers, including five female and five male speakers. Intra-observer reliability was quantified as the mean absolute difference between the original and repeated measurements. Mean absolute differences were 25.76 Hz for F1 and 30.66 Hz for F2. Mean signed differences were small (F1: −3.68 Hz; F2: 3.34 Hz), suggesting no systematic directional bias between measurement rounds.

Approximately 0.12–0.15 seconds of audio (corresponding to 10–12 video frames) was analyzed for each vowel. Vowel midpoints in utterances were used to reduce possible formant transition effects due to coarticulation. For the diphthongs in ‘bait’ and ‘boat’, we extracted the formants of the first vowel (e, o) at its midpoint. Definitive vowel identification was cross-validated with the articulatory patterns in the rtMRI image frames at concurrent timestamps. Mean fundamental frequency (F0) was measured for every speaker and every vowel similarly to formant extraction. Analogous to the morphometric data, the formant measurements from the two rounds of recordings were averaged to obtain one mean acoustic value for each vowel and each speaker.

Lastly, given the noise emitted by the MRI scanner, the acoustics of the speech recordings may contain features of the Lombard effect [80,81]. However, since the articulatory and acoustic data are recorded simultaneously, it is reasonable to assume that both data sets are similarly biased, such that morpho-phonetic covariation remains unbiased. Nevertheless, future studies may benefit from improving the sound quality of intra-MRI audio recordings toward more natural settings.

### Morpho-phonetic co-analysis

**Data preparation.** Sex and vocal tract size are known to be important factors influencing the speech signal [34,37,82,83]. Indeed, preliminary analyses of our morpho-phonetic data showed significant effects of sex and vocal tract size on vocal tract shape and acoustic output. In this study, we focused on those patterns of interindividual articulatory and acoustic variation that are independent of sex and vocal tract size and are thus common to all individuals in the sample. To remove the effects of sex and size from the data, we proceeded as follows. The vocal tract shape data (PCs 1–10) as well as the acoustic data (formants F1–F4 and fundamental frequency F0) were regressed against centroid size of the vocal tract and sex. The respective technique (multivariate multiple regression) is analogous to a multivariate analysis of covariance (MANCOVA) and was performed with the R packages *geomorph* and *RRPP* [77,84]. All subsequent analyses were then performed on the residuals of the regression data.

**Correlations between articulatory and phonetic distances.** Correlations were evaluated by regressing, for each individual, the *N* = 10 Euclidean distances between the five vowels in acoustic space versus articulatory space (Fig 1F). To compute the distances in acoustic space, we used formant wavelengths rather than frequencies. This is based on the consideration that the vocal tract acts as a resonating body, such that articulatory changes to its dimensions tend to elicit proportional changes in formant wavelengths rather than frequencies.

**Covariance between articulatory and acoustic data.** In both multidimensional articulatory and acoustic spaces, individual vowel polygons can be characterized by three properties: (a) location in space, as given by the coordinates of the polygon centroid (i.e., the average position of all vowels); (b) polygon size, quantified as its centroid size (the square root of the sum of the vowels’ squared distances from the centroid); and (c) polygon shape (the vowel coordinates relative to the centroid location). These geometric properties serve as proxies for the following real-life morphological and acoustic properties: in morphological terms, polygon location is a proxy for the resting-state morphology of the vocal tract (the average of all vowel articulations), polygon size corresponds to the articulatory range, and polygon shape characterizes the articulatory contrasts between vowels. In acoustic terms, polygon location corresponds to the timbre of the voice, size to the acoustic range, and shape to the acoustic contrasts between vowels.

Partial least-squares (PLS) analysis was used to study the covariation between the articulatory and acoustic vowel polygon data, and to explore and visualize how individual vocal tract shape influences the acoustic output. PLS permits the exploration of the covariation between two blocks of multidimensional data. Here, the multidimensional data are *N* × 2*P* matrices of articulatory landmark data (*N* = number of speakers, 2*P* = the 2D coordinates of *P* landmarks), *N* × 5*Z* matrices of articulatory data (*N* = number of speakers, 5*Z* = number of vowels × number of shape PCs) or *N* × 5*Q* matrices of acoustic data (*N* = number of speakers, 5*Q* = number of vowels × number of formants). We used PLS to evaluate the covariation between palate shape (*P*_p_ = 31) and tongue shape (*P*_t_ = 50) (Fig 2), and between the vowel polygons in articulatory space (5*Z* = 50) and in acoustic space (5*Q* = 10) (Fig 3). Similar to PCA, PLS is a dimension reduction technique. It uses singular value decomposition of the covariance matrix of two data blocks (i.e., data matrices) to extract pairs of orthogonal components (termed here PLS component pairs) that capture the largest, second-largest, etc., proportions of the total covariance in the sample. Due to the high dimensionality of the data, significance levels for PLS covariance (*p*_pls_) were estimated empirically with the permutation procedures (1,000 iterations) implemented in the R package *geomorph* [77]. We also evaluated the covariance ratio (CR) [85], which is a dimension-independent measure of the degree of association between two sets of multivariate data. The CR quantifies the covariance between two multidimensional data blocks as a fraction of the covariance within each block.

**Data visualization and rendering.** To visualize the correlated articulatory-acoustic (morpho-phonetic) variation, we proceeded as follows. For Fig 2, we evaluated the average tongue and palate shape of the three most extreme individuals at opposite ends of the data scatter (Fig 2A). To generate the vocal tract profiles (Fig 2C) of the two extremes, we calculated the distances between 21 tongue landmarks (tongue tip to tongue back) and the palate outline. Distances were measured perpendicular to the central axis of the air tube using ImageJ [86]. A similar approach was used for Fig 3, where the average vocal tract and formant configurations of the three individuals at each of the opposite ends of the data scatter were calculated (Fig 3A), and visualized in Fig 3A–3C.

### Cross-linguistic phonetic data (VC-C)

In order to compare the patterns of group-level morpho-phonetic variation in the MP-E sample with patterns of language-level variation, we used the VoxCommunis corpus (VC-C) [47], a large-scale cross-linguistic data set, which builds on a massively multilingual collection of transcribed speech from the publicly available Mozilla Common Voice Corpus [87]. The VC-C data set includes acoustic-phonetic measurements (F1–F4) of 36 different languages. From these, we selected the languages that included the five cardinal vowels (i, e, a, o, u) that we used in our morpho-phonetic analyses of the MP-E data. We excluded data with obvious errors or too few speakers. We analyzed data points only from speakers who produced the full set of vowels (i, e, a, o, u), in order to get the full vowel polygon of each speaker. This led to a sample of 19,335 data points (vowel utterances) from 3,867 individuals across 10 different languages (Basque, Bulgarian, Hindi, Italian, Portuguese, Romanian, Russian, Tamil, Turkish, Vietnamese), representing four language families (Indo-European, Dravidian, Turkic, Austroasiatic) and the language isolate Basque. We note limitations of this small language sample in terms of potential statistical bias from language genealogy and areal contact. The sample spans language splits at different depths of time and different degrees of geographical proximity, but remains too small to fully disentangle the effects of language genealogy, areal contact and cross-language influence. We therefore do not treat the VC-C sample as a phylogenetically or spatially independent estimate of global vowel diversity. Rather, we use it as a heterogeneous language sample from which a pattern of between-language vowel polygon variation can be estimated and compared with within-language variation. The languages do not form strongly separated genealogical or areal clusters in phonetic PC space (Fig 4B), supporting the use of this sample for formulating the neutral-like expectation of the biocultural model developed here.

A graphical comparison of the vowel-specific variation patterns in the MP-E and VC-C samples is presented in Fig 4A. This graph was constructed by correcting for source-specific offsets between the two data sets, which may arise from differences in recording conditions and formant-extraction procedures. The correction was applied separately for each vowel category and data source, but not separately by language or speaker group. Specifically, for each vowel, F1–F2 coordinates were translated so that the source-specific vowel centroid was superimposed onto the corresponding vowel-specific grand mean. This procedure therefore aligns vowel-specific distributions across data sources for graphical comparison only, while preserving the within-vowel scatter shown in Fig 4A.

Statistical comparisons of vowel polygon variation within and between language-specific samples are presented in Figs 4B and S9. These analyses were based on the unadjusted N×5Q vowel polygon data, where each observation represents a complete five-vowel polygon in acoustic space, with five vowels and *Q* = 2 formant coordinates (*N* = 3,867). We used the unadjusted coordinates so that vowel polygon location, size, shape, and between-vowel contrasts were retained for the variance-covariance analyses. Thus, the centroid-superimposed data were used only for the graphical comparison of per-vowel distributions in Fig 4A, whereas all statistical analyses of within- and between-language vowel polygon variation were performed on unadjusted vowel polygon coordinates. Furthermore, we ran data adjustment procedures similar to those used for the MP-E data in order to obtain sex-adjusted between-language data (pooled for sex, *N* = 3,867). Variation in the sex-pooled phonetic data was then decomposed into variation within languages (${𝐕}_{a.w}$), i.e., shared by all languages, and variation between languages (${𝐕}_{a.b}$). ${𝐕}_{a.w}$ was evaluated by pooling the data for language (*N* = 3,867), while ${𝐕}_{a.b}$ was evaluated from the mean data per language.

### Comparing patterns of variation

**Graphical comparison.** We compared the principal patterns of phonetic variation in the MP-E sample and in the VC-C sample (both within languages and between languages) by projecting all data into formant space (Fig 4A; variation per vowel) as well as into a common PC space (Fig 4B; variation of the vowel polygon). To construct the graph of Fig 4B, the vowel polygon data were expressed as data vectors of length 5*Q* (5 vowels, *Q* = 2 formants). PCA was performed on the 10×10 data matrix of the mean vowel polygons of the 10 languages, such that PC1 of Fig 4B expresses the principal direction of between-language variation of the vowel polygon. The principal directions of within-language variation in the VC-C and MP-E samples were projected into the same PC space. Finally, the principal direction of morpho-phonetic covariation in the MP-E sample (acoustic PLS1 in Fig 3A) was also projected into the PC space.

**Statistical comparison of variance-covariance structure.** The near-collinearity of the principal variation vectors in Fig 4B indicates proportionality of the variance-covariance matrices underlying these distributions. Statistical tests on proportionality were performed with eigenanalysis [88], as implemented in the R package *vcvcomp* [89] (see Vocal tract variation influences within- and between-language phonetic diversity in the main text).

**Test of the neutral expectation.** In analogy to the standard model of neutral phenotypic evolution, we expect that, under neutral conditions of language change, the pattern of vowel polygon variation between languages is proportional to the variation within languages, ${𝐕}_{a.b}\sim {𝐕}_{a.w}$ [54]. This proposition can be tested as follows [55]: the VCV (variance-covariance) matrices characterizing between-language and within-language variation are each orthogonalized, yielding statistically independent variance components along the eigenvectors. Then the between-group variance components are regressed against the within-group components. Proportionality between the two VCV matrices is indicated by linearity (i.e., slope = 1 in a double-logarithmic plot; S9 Fig).

## Supporting information

S1 Fig Example of rtMRI image frames during vowel production. The data underlying this Figure can be found in [43].
(TIFF)

S2 Fig Schematic representation of landmarks placed on 2D midsagittal images of the vocal tract. **(A)** Anatomical landmarks (reference points) are in red and semilandmarks in blue. **(B)** Landmark definitions. See S1 Table for detailed descriptions of the location of the landmarks.
(TIFF)

S3 Fig Articulatory shape variation associated with the PCs of shape space. **(A)** Shape variation along PCs 1 and 2. **(B)** Shape variation along PCs 1 and 3. **(C)** Shape variation along PCs 1 and 4. **(D)** Visualization of vocal tract shape variation along PCs 1–4 at the two ends of the respective data scatter. The data underlying this Figure can be found at https://osf.io/aeg7p/.
(TIFF)

S4 Fig Physical shape variation in the full sample of speakers producing the vowel [i]. The data underlying this Figure can be found at https://osf.io/aeg7p/.
(TIFF)

S5 Fig Physical shape variation in the full sample of speakers producing the vowel [e]. The data underlying this Figure can be found at https://osf.io/aeg7p/.
(TIFF)

S6 Fig Physical shape variation in the full sample of speakers producing the vowel [a]. The data underlying this Figure can be found at https://osf.io/aeg7p/.
(TIFF)

S7 Fig Physical shape variation in the full sample of speakers producing the vowel [o]. The data underlying this Figure can be found at https://osf.io/aeg7p/.
(TIFF)

S8 Fig Physical shape variation in the full sample of speakers producing the vowel [u]. The data underlying this Figure can be found at https://osf.io/aeg7p/.
(TIFF)

S9 Fig Regression of between-group (between-language) variances on within-group variances. **(A)** Regression of between-language variance on within-group variance by means of PCs 1–10 of vowel polygon form in F1–F2 formant space. The slope does not deviate significantly from 1 (*p* = 0.684). The within-group variances were computed on the MP-E data and the VC-C data. **(B)** Separate regression for each VC-C language (each color corresponds to a different language) and the MP-E (black dashed line). The data underlying this Figure can be found at https://osf.io/aeg7p/.
(TIFF)

S1 Table Orofacial landmarks (LM) used in this study. Landmark identifiers (ID) as in S2 Fig. K: number of semilandmarks per curve; Type: fixed landmark (F), curve semilandmark (C); Tissue: soft (S), hard (H).
(CSV)

S2 Table Variance ratios of within-vowel variance versus between-vowel variance. (CSV)

## Abbreviations

CR: covariance ratio
GM: geometric morphometrics
GPA: Generalized Procrustes Analysis
MP-E: Morpho-phonetic data of English speakers
PCA: Principal Components Analysis
PLS: partial least-squares analysis
rtMRI: real-time Magnetic Resonance Imaging
VC-C: VoxCommunis Corpus
