# Supplementary material for: Interindividual vocal tract diversity influences the phonetic diversification of spoken languages
Source: PLoS Biol. 2026 Oct 13;24(10):e3004003. doi: 10.1371/journal.pbio.3004003 (PMC13660918; doi:10.1371/journal.pbio.3004003)
Supplement: Supplementary file 1 [file pbio.3004003.s012.pdf]

Dear Dr Moran,

Thank you for your patience while your manuscript "Interindividual vocal tract diversity influences the phonetic diversification of spoken languages" was peer-reviewed at PLOS Biology. It has now been evaluated by the PLOS Biology editors, an Academic Editor with relevant expertise, and by two independent reviewers.

You'll see that Reviewer #1 is impressed by the analysis, but remains sceptical that you have demonstrated “neutral processes” (or can indeed address *any* processes). S/he thinks that multiple alternative explanations have not been excluded. Reviewer #2 is more positive; s/he explicitly says that responses to the reviewers from the previous journal appear reasonable. S/he has a couple of textual points, but also says that s/he is not qualified to assess the pooling strategies used in your stats pipeline.

Given the somewhat divergent assessments from the two reviewers, I discussed these comments with the Academic Editor, who felt that you should be given an opportunity to address the reviewers' concerns, though they asked me to be firm in my insistence that where it is not possible to fully allay a concern, clear explanations should be given and limitations and/or alternative explanations clearly stated.

In light of the reviews, which you will find at the end of this email, we would like to invite you to revise the work to thoroughly address the reviewers' reports.

Given the extent of revision needed, we cannot make a decision about publication until we have seen the revised manuscript and your response to the reviewers' comments. Your revised manuscript is likely to be sent for further evaluation by all or a subset of the reviewers.

Sincerely,

Roli Roberts

Roland Roberts, PhD
Senior Editor
PLOS Biology

REVIEWERS' COMMENTS:

Reviewer #1:

This manuscript aims to shed light on the diversification of vowels in human languages.

To this end, the authors first conducted a technically advanced study of variation in vocal output characteristics between speakers and their relation to vocal tract morphology within one language. They analyzed a set of different vowels produced across a range of target words (two renditions per speaker) from 16 male and 16 female participants during real-time MRI imaging, which allows tracing variation in vocal tract configuration alongside acoustic variation. The authors found a good correlation between the acoustic characteristics derived from the 1st-to-2nd formant ratio and morphology, but also substantial variation within the speaker community. They argue that this variation could play a role in the evolution of different languages. Moreover, they stress that previous analyses have neglected this acoustic variation and treated it solely as noise. They then compare the variation within the examined language (American English) with that of ten other languages from the VoxCommunis corpus (VC-C) and report a strong correlation between within-language and between-language variation in F1-F2 space. They conclude that this variation points to a neutral-like evolutionary process.

> We thank the reviewer for their positive feedback and their detailed comments on our manuscript, which helped us clarify various open questions. The reviewer's critical comments encouraged us to develop a formal biocultural model of phonetic diversification (see updated discussion), which addresses all the specific questions asked by the reviewer.

The reported correlation between within- language variation and between-language divergence in F1-F2 space is an interesting and potentially informative result. I also commend the authors on their ambitious and well-documented analysis. However, I am not convinced that the reported pattern, by itself, constitutes strong evidence for a "neutral-like" diversification process. Indeed, the paper does not directly address potential processes, but focuses primarily on the reported patterns of variation.

> The first part of our paper (morpho-phonetic covariation in the MP-E sample) takes a process-oriented approach, showing that interindividual vocal tract variation is not fully compensated for by articulatory adaptation, resulting in a characteristic pattern of residual acoustic vowel variation among the individuals of our MP-E sample. To put it somewhat colloquially: Listeners can “hear” the shape of the speaker's vocal tract to a certain extent.

Generalizing these findings to additional language samples currently presents a significant methodological challenge: the VoxCommunis data is acoustic only and lacks an articulatory counterpart. Therefore, we agree with the reviewer that the second part of our manuscript, which analyzes acoustic covariation within and between languages, is more exploratory than confirmatory, as we identify patterns, but not processes. This is precisely why we use the term "neutral-like" (rather than "neutral"). We have identified a pattern of correlated variation in vowel acoustics that resembles the pattern expected under a neutral evolutionary process. However, as the reviewer rightly points out, we cannot rule out equifinality—for example, selection processes resulting in neutral-like patterns of vowel change among languages. We address this point in detail in our subsequent responses.

In response to the reviewer's comments, the revised manuscript now more clearly explains the logical connection between Parts 1 (morpho-phonetics) and 2 (acoustic variation within and across languages):

a) We demonstrate that vocal tract variation in the MP-E sample results in residual (non-compensated) acoustic variation, i.e., morpho-phonetic covariation.
b) We observe similar patterns of acoustic variation in the VC-C language samples and hypothesize that these patterns reflect morpho-phonetic covariation in all language samples.
c) We observe correlated patterns of within- and between-group variation in vowel acoustics and propose the "neutral-like" biocultural model of phonetic diversification as a null hypothesis to explain the correlation.

We have made these points clearer in the Discussion, as denoted by red text.

A positive association between internal variability and cross-language differences is certainly consistent with stochastic change. At the same time, it is important to note that such alignment is not specific to neutral processes. Vowel systems evolve within a highly structured acoustic-perceptual space. Because F1-F2 space is shaped by articulatory constraints and perceptual discriminability, some directions of variation are more accessible than others. Both adaptive pressures (e.g., maintaining contrast or optimizing dispersion) and internally biased sound change processes could therefore generate divergence that follows the same axes along which languages already exhibit greater internal variability.

We agree with the reviewer on this point. Our biocultural model of phonetic diversification does not presuppose neutrality, but actually incorporates the properties of the highly structured acoustic-perceptual space. In our morpho-phonetic PLS analyses,

we assume that the multivariate acoustic variance-covariance matrix V_a is the sum of the multivariate vocal tract variance-covariance matrix V_v and a term V_e, representing external influences and stochasticity:

$$V_a = V_v + V_e,$$

V_v thus quantifies how vocal tract shape and articulatory adaptation together influence the phonetic output. Importantly, V_a represents the covariance structure of all five vowels together (the vowel polygon's position, size and shape). It is a non-isotropic matrix that reflects, in the reviewer's words, the structure of the acoustic-perceptual space and all the internal biases of joint vowel variation. Likewise, V_v is non-isotropic and represents vocal tract variation among individuals as well as variation associated with the specific articulatory gestures used to produce the five vowels.

In our biocultural model of phonetic diversification at the level of speaker groups, the speakers' vocal tracts act as a source that constantly injects variation into the acoustic communication system, while the listeners' auditory system acts as a filter removing variation that potentially blurs vowel discrimination. We can now use Lande's equation of phenotypic evolutionary change under selection β to describe how our acoustic system evolves over time (Lande 1979, *Evolution* 33: 402-416)[1]. Over a given time interval (e.g. one generation), a language's vowel constellation (PC graph of Figure 4B) is expected to change by a quantity Δz:

$$\Delta z = V_a \beta,$$

where β is a selection-gradient vector that defines the listener filter (the "selection" for vowel discrimination). Now consider the log-log slope of between- versus within-group variation (Figure S9). Under a neutral scenario, the slope is expected to be 1. Assuming β acts pervasively on all PCs in the form of stabilizing selection (the reviewer's "highly structured acoustic-perceptual space"), it will not affect the slope of 1, but will proportionally shrink both the within- and between-group variances. This is why we use the term "neutral-like" rather than "neutral" to characterize our model.

We have clarified these points in the Discussion section.

Moreover, sound change is not isotropic. Well-documented tendencies such as coarticulatory effects introduce directional biases in how vowels move in acoustic space. Even without strong

[1] https://www.jstor.org/stable/2407630

external selection, such structured change can produce systematic divergence patterns that scale with within-language covariance.

> See above comment and equations. Indeed, acoustic variation, as expressed by V_a is structured and non-isotropic, and the direction of change Δz is therefore shaped by its axes of variation. The first eigenvector (PC axis) of V_a captures the direction of greatest variation, thus of least evolutionary resistance (the reviewer's "directional bias" in the five-vowel system). Neutral evolutionary models thus do not presuppose isotropy – neither at the level of genetics (not considered here) nor phenetics (the vocal tract) nor phonetics (the acoustic output). They just make predictions about how $V_{a.b}$ (between-group covariance matrix) evolves as a function of $V_{a.w}$ (within-group covariance matrix):

$$V_{a.b} = V_{a.w}\, t/N_e,$$

> where t is the time in generations, and N_e is the effective population size (in our case: all speakers). Under neutrality, $V_{a.b}$ is expected to increase linearly with time, i.e., speaker groups drift apart randomly.

> One of our manuscripts (under review) addresses the question of coarticulation more specifically. It shows that patterns of coarticulatory vowel sound variation differ substantially from the patterns observed here.[2]

A further issue concerns genealogical relationships. Closely related languages may resemble each other in both their covariance structure and their mean vowel positions due to shared history. Without explicitly addressing phylogenetic or spatial non-independence, it is difficult to determine whether the observed correlation reflects an ongoing diversification process or inherited structure.

> The reviewer points out a major limitation of our study, which has both methodological and logical causes. Phylogenetic non-independence could, in principle, be addressed using methods such as phylogenetic independent contrasts (PIC). However, PIC computation relies on an a priori evolutionary process model, usually the Brownian model of trait evolution (i.e., neutral evolution) or the Ornstein-Uhlenbeck model (which incorporates selection scenarios). In any case, the application of PIC would result in circularities, such as testing for ongoing neutral diversification using a neutral diversification model. We explicitly take this logical constraint into account by presenting our "neutral-like" biocultural model of phonetic diversification as a null hypothesis for testing patterns of language diversification.

[2] An earlier version of this manuscript is available here (chapter 3): https://libra.unine.ch/handle/20.500.14713/99596.

The reviewer rightly points out that addressing spatial non-independence (e.g., migration and cross-language influences) is as important as addressing phylogenetic non-independence. However, little is known about the duration and magnitude of such influences. This means that model specifications (e.g. via analogs of migration matrices) remain largely exploratory rather than explanatory.

In the revised Methods text (in red), we acknowledge these limitations and explain how we address them. We use a language sample representing language splits at various depths of time and degrees of geographical proximity. The pattern of between-group variation emerging from these data gives rise to the null hypothesis of "neutral-like" evolution.

In sum, the correlation between within- and between-language variation demonstrates compatibility with a neutral-like account, but it does not uniquely support it. To substantiate a neutral interpretation, the analysis would need to show that alternative explanations—such as constrained adaptive divergence, biased sound change, or shared ancestry—are unlikely to account for the observed pattern.

Our comments above report on how we dealt with constraints and biases. Essentially, constraints and biases are integral to Lande's classical neutral model of trait evolution.

Shared ancestry needs to be addressed in further studies based on large phylogenetically-controlled cross-linguistic samples, bearing in mind that the neutral-like hypothesis is part of the phylogenetic model rather than the results.

We have made this clearer in the manuscript, as already addressed in the comments above.

In addition, I found the authors somewhat dismissive of prior work on within-language variation in speech perception. There is ample evidence that listeners can recognize phonemes spoken by different speakers despite substantial variation among individuals (with body size variation being a well-known issue).

We apologize for any apparent dismissiveness toward earlier studies. In the revised text, we use more precise phrasing and clarify that, in listener-centered studies (such as automated language recognition), variation between speakers is usually accounted for, though it is not treated as data per se, but rather as noise.

A positive within-between variance correlation is predicted under drift, but it is also predicted under directional selection constrained by the covariance structure. The authors should be more cautious in their interpretation.

> We agree with the reviewer that within-between population variance correlations are positive, both under scenarios of selection and drift. Positive correlations and slopes basically indicate that populations tend to diversify along the same directions of variation along which individuals vary within populations. (Negative slopes would indicate that populations diverge along directions of least within-group variation, which is highly improbable.) Nevertheless, it is important to note that the slope of the log-log variance plot (our Figure S9) permits discriminating between different diversifying scenarios (cf. Ackermann, R. R., & Cheverud, J. M.; 2004)[3]. We have therefore revised the Discussion to clarify that a slope of one is the neutral expectation, whereas deviations from one suggest that perceptual, social, cultural, environmental, historical, or other selective forces act differentially across axes of vowel-polygon variation.

Minor points, per line

16: "environment, culture, lifestyle shape an individual's vocal tract" -- Blasi et al. discuss changes in vocal tract morphology since the Neolithic. The statement is somewhat misleading as it suggests that current eating habits shape one's vocal tract. The references given (general books on the evolution of language plus the Blasi paper) do not provide evidence for such a notion.

> We have updated this parenthetical to "(developmental and sociocultural factors)" and updated the references accordingly.

24: "how phonetic variation within speaker communities gives rise to variation between communities, and eventually between languages" suggests that the authors are studying the PROCESSES giving rise to variation between languages, but that is not correct. See also line 135: the authors do not study diversification (implying the process).

> Here are the two full sentences for convenience and how we have rewritten them:
>
> 24: Here we focus on the biological side of the question, asking specifically how vocal tract variation within a speaker group influences phonetic variation, and how phonetic variation within speaker communities gives rise to variation between communities, and eventually between languages.

[3] https://www.pnas.org/doi/abs/10.1073/pnas.0405919102

Updated to:

Here we focus on the biological side of the question, asking specifically how vocal tract variation within a speaker group influences phonetic variation, and how phonetic variation within speaker groups correlates with variation between groups.

And:

135: As a complement, we take a bottom-up approach to track how individual vocal tract variation in a speaker group serves as a source of articulatory and phonetic variation, and how phonetic variation within groups serves as a source of phonetic diversification between groups.

Updated to:

As a complement to such top-down approaches, we have taken a bottom-up approach here that starts with a focus on individual morpho-phonetic features. While these features are statistically independent of sex and vocal tract size (see Methods), we have shown that they carry significant information about the shape of an individual's vocal tract. We argue here that the underlying pattern of interindividual vocal tract variation is not only a source of phonetic variation within speaker groups, but also a potential source of phonetic variation and diversification between groups. To formalize this idea, we propose a biocultural model of phonetic diversification, by analogy to Lande's well-established biological model of multivariate phenotypic evolution (Lande, 1979).

125: What is a common mode of vocal tract variation?

We agree that the phrase "common mode of vocal tract variation" was unclear. We intended to refer to a recurrent pattern of speaker-level phonetic variation that, based on the MP-E data, is plausibly grounded in shared vocal tract constraints. The phrase "common mode" also appeared in the Figure 4 caption. We have revised the sentence in the results to:

Since the MP-E phonetic variation is conditioned by vocal tract variation (Fig. 3A), we infer that there exists a basic pattern of speaker-level phonetic variation within languages that is grounded, at least in part, in the constraints imposed by vocal tract variation among speakers.

And we have revised "common mode" in the Fig. 4 caption to: "*shared pattern*".

192: How did the authors deal with the non-independence of the vowels (two per speaker) in subsequent statistical analyses? On line 214, they state that a mean configuration was calculated from the MRI data. Was the same true for the vowels?

> We thank the reviewer for raising this point. The two repetitions of each vowel were not treated as independent observations. For both the morphometric and acoustic data, repeated measurements were averaged to obtain one mean observation for each vowel and each speaker. Thus, the final MP-E data set consisted of 5 vowels x 32 speakers = 160 speaker-by-vowel observations. We have clarified this in the Methods (text in red) by explicitly stating that the acoustic measurements were averaged in the same way as the rtMRI configurations.

225: Visual inspection: How high was the observer reliability?

> We thank the reviewer for pointing this out. We have now added an explicit assessment of intra-observer reliability to the Methods section.

Reviewer #2:

Plos Biol 2026 Review

The authors of this manuscript offer an interesting and novel perspective on the variation observed in speech sounds. Specifically, they focus on less addressed, biological considerations, asking 'how vocal tract variation within a speaker community influences phonetic variation, and how phonetic variation within speaker communities gives rise to variation between communities, and eventually between languages'. Through several analyses, they offer a thorough, step-wise journey detailing the impact of vocal tract anatomy on the use of articulators, and the variation of each of these on subsequent vowel sounds produced.

They use neat methods, focusing on the five cardinal vowels and select 32 speakers from different language families which specifically use these vowels. They use a new approach which combines geometric morphometric vocal tract analysis using real time MRI with spectral (formant) analysis of the simultaneous audio signal emitted whilst pronouncing vowels. These are then sampled with synchronous time stamps.

Particularly interesting is the finding that a wide variety of articulatory gestures eg using lips, tongue, velum result in much smaller acoustic variation in vowel shape. The suggestion is that

this supports earlier evidence that individuals adapt their articulatory gestures to produce similar sounding vowels.

An additional analysis then assesses if variation in fixed anatomical features may have a causal effect here, specifically the palate shape partially accounts for tongue shape variation, such that individuals with short, rounded hard palates produce more rounded tongue gestures than individuals with elongated and shallow palates. However, articulations do not fully compensate for acoustic variation, such that some remains and can be acoustically identified.

I see this manuscript has already been reviewed once and the response to the reviewers is included - these responses make sense to me so I will mainly not revisit these points. Pending a point made below about the stats, my other points are very minor. I see this research is innovative, insightful , of inter-disciplinary interest and highly relevant for this journal and its readers.

> We thank the reviewer for their positive feedback and these insightful comments.

Analyses

For some analyses, cross-linguistic phonetic data, data were subjected to a number of treatments:

L.302-308. To directly compare the VC-C data with our MP-E data, we adjusted the data for differences in formant extraction methods by superimposing vowel centroids. Furthermore, we ran similar data adjustment procedures as for the MP-E in order to obtain sex-adjusted between-language data (pooled for sex, N=19,335). Variation in the sex-pooled phonetic data was then decomposed into variation within languages (Vpho.w), i.e., shared by all languages and variation between languages (Vpho.b). Vpho.w was evaluated by pooling the data for language (N=19,335), while Vpho.b was evaluated from the mean data per language.

Whilst the statistical approach seems thorough, interesting and logical, it is beyond my stats knowledge to be able to assess the full impact of all statistical treatments through the whole 'pipeline', for example why pooling versus not pooling data at various steps is necessary or helpful. If not already done, a specialist reviewer is advised here.

> We thank the reviewer for this thoughtful comment. We agree that the analysis pipeline involves several treatment steps, and we therefore clarify the rationale here. The purpose of the morpho-phonetic co-analysis was not to analyze raw formant variation directly, but to isolate the component of interindividual and cross-linguistic phonetic variation that is

comparable across speakers and languages after accounting for major known sources of variation.

For the MP-E data, sex and vocal tract size were treated as covariates because both are known to strongly influence vocal tract shape and the speech signal. We therefore regressed the articulatory shape variables and acoustic variables against sex and centroid size using multivariate multiple regression, which follows the same statistical rationale as MANCOVA[4,5]. Subsequent analyses were performed on the residuals. This is a standard approach when the aim is to analyze multivariate variation after accounting for the effects of specified covariates. The resulting residuals therefore represent the component of morpho-phonetic variation not explained by sex or overall vocal tract size.

For the VC-C data, analogous adjustments were applied to make the cross-linguistic acoustic data comparable to the MP-E data. Superimposing vowel centroids reduces global offsets between data sets that may arise from differences in recording conditions or formant extraction procedures, while preserving the relative geometry of vowel configurations. Sex adjustment reduced a major source of speaker-level formant variation. The subsequent pooling steps separated different levels of variation: pooling individual observations across languages estimated recurrent within-language phonetic variation, whereas language means estimated between-language variation. Comparing these two components allowed us to test whether the multivariate structure of variation within languages resembles the structure of diversification between languages.

Thus, the treatments were not intended to alter the biological signal, but to remove known confounding effects and to separate within-language from between-language components of variation in a statistically controlled way.

To make this more clear in the manuscript we edited the Materials and Methods section (text changes in red).

Results

L.127-130. Just because the variation is similar within and between languages does not have to mean that the same processes govern both. At least there is a lot more possibilities inherent in this statement than seems to be presented in this paragraph. It would be helpful to clarify how such processes may be operating within and between languages that could lead to phonetic diversification language diversification. This is a fascinating point and well worth expansion.

---

[4] https://link.springer.com/article/10.1007/s11692-009-9055-x
[5] https://besjournals.onlinelibrary.wiley.com/doi/full/10.1111/2041-210X.13029

In line with R1’s comments regarding directional and stabilizing selection (both from an evolutionary perspective and from that of linguistic and socio-cultural factors), we have updated our Discussion section. See red text in manuscript and comments above.

Discussion

This is clearly fascinating - and brings to mind how this might compare with other species - how variation in other species' vocal tracts and downstream impact on variation within species vocalisations. In my view, since there goal of the paper relates to a biological rather than linguistic perspective, adding at least in the discussion, a nod in the direction of comparison with other species could broaden the scope of findings.

We agree and we have added some text to the end of the Discussion to highlight this point, and the points also raised by R1.
