# Supplementary material for: Interindividual vocal tract diversity influences the phonetic diversification of spoken languages
Source: PLoS Biol. 2026 Oct 13;24(10):e3004003. doi: 10.1371/journal.pbio.3004003 (PMC13660918; doi:10.1371/journal.pbio.3004003)
Supplement: Peer review history

# Editor report: Decision Letter 0

**DOI:** 10.1371/journal.pbio.3004003.r001

**Authors:** Roland G Roberts

11 Feb 2026

Dear Dr Moran,

Thank you for submitting your manuscript entitled "Interindividual vocal tract diversity influences thephonetic diversification of spoken languages" for consideration as a Research Article by PLOS Biology.

Your manuscript has now been evaluated by the PLOS Biology editorial staff, as well as by an academic editor with relevant expertise, and I'm writing to let you know that we would like to send your submission out for external peer review. Please accept my apologies for the delay incurred while we sought external expert advice.

However, before we can send your manuscript to reviewers, we need you to complete your submission by providing the metadata that is required for full assessment. To this end, please login to Editorial Manager where you will find the paper in the 'Submissions Needing Revisions' folder on your homepage. Please click 'Revise Submission' from the Action Links and complete all additional questions in the submission questionnaire.

Once your full submission is complete, your paper will undergo a series of checks in preparation for peer review. After your manuscript has passed the checks it will be sent out for review. To provide the metadata for your submission, please Login to Editorial Manager (https://www.editorialmanager.com/pbiology) within two working days, i.e. by Feb 13 2026 11:59PM.

During the process of completing your manuscript submission, you will be invited to opt-in to posting your pre-review manuscript as a bioRxiv preprint. Visit http://journals.plos.org/plosbiology/s/preprints for full details. If you consent to posting your current manuscript as a preprint, please upload a single Preprint PDF.

Feel free to email us at plosbiology@plos.org if you have any queries relating to your submission.

Kind regards,

Roli Roberts

Roland Roberts, PhD

Senior Editor

PLOS Biology

rroberts@plos.org

# Peer review: Decision Letter 1

**DOI:** 10.1371/journal.pbio.3004003.r002

**Authors:** Roland G Roberts

24 Apr 2026

Dear Dr Moran,

Thank you for your patience while your manuscript "Interindividual vocal tract diversity influences the phonetic diversification of spoken languages" was peer-reviewed at PLOS Biology. It has now been evaluated by the PLOS Biology editors, an Academic Editor with relevant expertise, and by two independent reviewers.

You'll see that Reviewer #1 is impressed by the analysis, but remains sceptical that you have demonstrated “neutral processes” (or can indeed address *any* processes). S/he thinks that multiple alternative explanations have not been excluded. Reviewer #2 is more positive; s/he explicitly says that responses to the reviewers from the previous journal appear reasonable. S/he has a couple of textual points, but also says that s/he is not qualified to assess the pooling strategies used in your stats pipeline.

Given the somewhat divergent assessments from the two reviewers, I discussed these comments with the Academic Editor, who felt that you should be given an opportunity to address the reviewers' concerns, though they asked me to be firm in my insistence that where it is not possible to fully allay a concern, clear explanations should be given and limitations and/or alternative explanations clearly stated.

In light of the reviews, which you will find at the end of this email, we would like to invite you to revise the work to thoroughly address the reviewers' reports.

Given the extent of revision needed, we cannot make a decision about publication until we have seen the revised manuscript and your response to the reviewers' comments. Your revised manuscript is likely to be sent for further evaluation by all or a subset of the reviewers.

In addition to these revisions, you will need to complete some formatting changes, which you will receive in a follow up email. A member of our team will be in touch with a set of requests shortly.

We expect to receive your revised manuscript within 3 months. Please email us (plosbiology@plos.org) if you have any questions or concerns, or would like to request an extension.

At this stage, your manuscript remains formally under active consideration at our journal; please notify us by email if you do not intend to submit a revision so that we may withdraw it.

**IMPORTANT - SUBMITTING YOUR REVISION**

Your revisions should address the specific points made by each reviewer. Please submit the following files along with your revised manuscript:

1. A 'Response to Reviewers' file - this should detail your responses to the editorial requests, present a point-by-point response to all of the reviewers' comments, and indicate the changes made to the manuscript.

*NOTE: In your point-by-point response to the reviewers, please provide the full context of each review. Do not selectively quote paragraphs or sentences to reply to. The entire set of reviewer comments should be present in full and each specific point should be responded to individually, point by point.

You should also cite any additional relevant literature that has been published since the original submission and mention any additional citations in your response.

2. In addition to a clean copy of the manuscript, please also upload a 'track-changes' version of your manuscript that specifies the edits made. This should be uploaded as a "Revised Article with Changes Highlighted" file type.

*Re-submission Checklist*

When you are ready to resubmit your revised manuscript, please refer to this re-submission checklist: https://plos.io/Biology_Checklist

To submit a revised version of your manuscript, please go to https://www.editorialmanager.com/pbiology/ and log in as an Author. Click the link labelled 'Submissions Needing Revision' where you will find your submission record.

Please make sure to read the following important policies and guidelines while preparing your revision:

*Published Peer Review*

Please note while forming your response, if your article is accepted, you may have the opportunity to make the peer review history publicly available. The record will include editor decision letters (with reviews) and your responses to reviewer comments. If eligible, we will contact you to opt in or out. Please see here for more details:

https://blogs.plos.org/plos/2019/05/plos-journals-now-open-for-published-peer-review/

*PLOS Data Policy*

Please note that as a condition of publication PLOS' data policy (http://journals.plos.org/plosbiology/s/data-availability) requires that you make available all data used to draw the conclusions arrived at in your manuscript. If you have not already done so, you must include any data used in your manuscript either in appropriate repositories, within the body of the manuscript, or as supporting information (N.B. this includes any numerical values that were used to generate graphs, histograms etc.). For an example see here: http://www.plosbiology.org/article/info%3Adoi%2F10.1371%2Fjournal.pbio.1001908#s5

*Protocols deposition*

To enhance the reproducibility of your results, we recommend that if applicable you deposit your laboratory protocols in protocols.io, where a protocol can be assigned its own identifier (DOI) such that it can be cited independently in the future. Additionally, PLOS ONE offers an option for publishing peer-reviewed Lab Protocol articles, which describe protocols hosted on protocols.io. Read more information on sharing protocols at https://plos.org/protocols?utm_medium=editorial-email&utm_source=authorletters&utm_campaign=protocols

Thank you again for your submission to our journal. We hope that our editorial process has been constructive thus far, and we welcome your feedback at any time. Please don't hesitate to contact us if you have any questions or comments.

Sincerely,

Roli Roberts

Roland Roberts, PhD

Senior Editor

PLOS Biology

rroberts@plos.org

------------------------------------

REVIEWERS' COMMENTS:

Reviewer #1:

This manuscript aims to shed light on the diversification of vowels in human languages.

To this end, the authors first conducted a technically advanced study of variation in vocal output characteristics between speakers and their relation to vocal tract morphology within one language. They analyzed a set of different vowels produced across a range of target words (two renditions per speaker) from 16 male and 16 female participants during real-time MRI imaging, which allows tracing variation in vocal tract configuration alongside acoustic variation. The authors found a good correlation between the acoustic characteristics derived from the 1st-to-2nd formant ratio and morphology, but also substantial variation within the speaker community. They argue that this variation could play a role in the evolution of different languages. Moreover, they stress that previous analyses have neglected this acoustic variation and treated it solely as noise. They then compare the variation within the examined language (American English) with that of ten other languages from the VoxCommunis corpus (VC-C) and report a strong correlation between within-language and between-language variation in F1-F2 space. They conclude that this variation points to a neutral-like evolutionary process.

The reported correlation between within- language variation and between-language divergence in F1-F2 space is an interesting and potentially informative result. I also commend the authors on their ambitious and well-documented analysis. However, I am not convinced that the reported pattern, by itself, constitutes strong evidence for a "neutral-like" diversification process. Indeed, the paper does not directly address potential processes, but focuses primarily on the reported patterns of variation.

A positive association between internal variability and cross-language differences is certainly consistent with stochastic change. At the same time, it is important to note that such alignment is not specific to neutral processes. Vowel systems evolve within a highly structured acoustic-perceptual space. Because F1-F2 space is shaped by articulatory constraints and perceptual discriminability, some directions of variation are more accessible than others. Both adaptive pressures (e.g., maintaining contrast or optimizing dispersion) and internally biased sound change processes could therefore generate divergence that follows the same axes along which languages already exhibit greater internal variability.

Moreover, sound change is not isotropic. Well-documented tendencies such as coarticulatory effects introduce directional biases in how vowels move in acoustic space. Even without strong external selection, such structured change can produce systematic divergence patterns that scale with within-language covariance.

A further issue concerns genealogical relationships. Closely related languages may resemble each other in both their covariance structure and their mean vowel positions due to shared history. Without explicitly addressing phylogenetic or spatial non-independence, it is difficult to determine whether the observed correlation reflects an ongoing diversification process or inherited structure.

In sum, the correlation between within- and between-language variation demonstrates compatibility with a neutral-like account, but it does not uniquely support it. To substantiate a neutral interpretation, the analysis would need to show that alternative explanations—such as constrained adaptive divergence, biased sound change, or shared ancestry—are unlikely to account for the observed pattern.

In addition, I found the authors somewhat dismissive of prior work on within-language variation in speech perception. There is ample evidence that listeners can recognize phonemes spoken by different speakers despite substantial variation among individuals (with body size variation being a well-known issue).

A positive within-between variance correlation is predicted under drift, but it is also predicted under directional selection constrained by the covariance structure. The authors should be more cautious in their interpretation.

Minor points, per line

16: "environment, culture, lifestyle shape an individual's vocal tract" -- Blasi et al. discuss changes in vocal tract morphology since the Neolithic. The statement is somewhat misleading as it suggests that current eating habits shape one's vocal tract. The references given (general books on the evolution of language plus the Blasi paper) do not provide evidence for such a notion.

24: "how phonetic variation within speaker communities gives rise to variation between communities, and eventually between languages" suggests that the authors are studying the PROCESSES giving rise to variation between languages, but that is not correct. See also line 135: the authors do not study diversification (implying the process).

125: What is a common mode of vocal tract variation?

192: How did the authors deal with the non-independence of the vowels (two per speaker) in subsequent statistical analyses? On line 214, they state that a mean configuration was calculated from the MRI data. Was the same true for the vowels?

225: Visual inspection: How high was the observer reliability?

Reviewer #2:

Plos Biol 2026 Review

The authors of this manuscript offer an interesting and novel perspective on the variation observed in speech sounds. Specifically, they focus on less addressed, biological considerations, asking 'how vocal tract variation within a speaker community influences phonetic variation, and how phonetic variation within speaker communities gives rise to variation between communities, and eventually between languages'. Through several analyses, they offer a thorough, step-wise journey detailing the impact of vocal tract anatomy on the use of articulators, and the variation of each of these on subsequent vowel sounds produced.

They use neat methods, focusing on the five cardinal vowels and select 32 speakers from different language families which specifically use these vowels. They use a new approach which combines geometric morphometric vocal tract analysis using real time MRI with spectral (formant) analysis of the simultaneous audio signal emitted whilst pronouncing vowels. These are then sampled with synchronous time stamps.

Particularly interesting is the finding that a wide variety of articulatory gestures eg using lips, tongue, velum result in much smaller acoustic variation in vowel shape. The suggestion is that this supports earlier evidence that individuals adapt their articulatory gestures to produce similar sounding vowels.

An additional analysis then assesses if variation in fixed anatomical features may have a causal effect here, specifically the palate shape partially accounts for tongue shape variation, such that individuals with short, rounded hard palates produce more rounded tongue gestures than individuals with elongated and shallow palates. However, articulations do not fully compensate for acoustic variation, such that some remains and can be acoustically identified.

I see this manuscript has already been reviewed once and the response to the reviewers is included - these responses make sense to me so I will mainly not revisit these points. Pending a point made below about the stats, my other points are very minor. I see this research is innovative, insightful , of inter-disciplinary interest and highly relevant for this journal and its readers.

Analyses

For some analyses, cross-linguistic phonetic data, data were subjected to a number of treatments:

L.302-308. To directly compare the VC-C data with our MP-E data, we adjusted the data for differences in formant extraction methods by superimposing vowel centroids. Furthermore, we ran similar data adjustment procedures as for the MP-E in order to obtain sex-adjusted between-language data (pooled for sex, N=19,335). Variation in the sex-pooled phonetic data was then decomposed into variation within languages (Vpho.w), i.e., shared by all languages and variation between languages (Vpho.b). Vpho.w was evaluated by pooling the data for language (N=19,335), while Vpho.b was evaluated from the mean data per language.

Whilst the statistical approach seems thorough, interesting and logical, it is beyond my stats knowledge to be able to assess the full impact of all statistical treatments through the whole 'pipeline', for example why pooling versus not pooling data at various steps is necessary or helpful. If not already done, a specialist reviewer is advised here.

Results

L.127-130. Just because the variation is similar within and between languages does not have to mean that the same processes govern both. At least there is a lot more possibilities inherent in this statement than seems to be presented in this paragraph. It would be helpful to clarify how such processes may be operating within and between languages that could lead to phonetic diversification language diversification. This is a fascinating point and well worth expansion.

Discussion

This is clearly fascinating - and brings to mind how this might compare with other species - how variation in other species' vocal tracts and downstream impact on variation within species vocalisations. In my view, since there goal of the paper relates to a biological rather than linguistic perspective, adding at least in the discussion, a nod in the direction of comparison with other species could broaden the scope of findings.

# Author comment: Author response to Decision Letter 2

**DOI:** 10.1371/journal.pbio.3004003.r003

23 Jul 2026

Attachment Submitted filename: response_to_reviewers.pdf

# Peer review: Decision Letter 2

**DOI:** 10.1371/journal.pbio.3004003.r004

**Authors:** Roland G Roberts

21 Aug 2026

Dear Dr Moran,

Thank you for your patience while we considered your revised manuscript "Interindividual vocal tract diversity influences the phonetic diversification of spoken languages" for publication as a Research Article at PLOS Biology. This revised version of your manuscript has been evaluated by the PLOS Biology editors, the Academic Editor and the original reviewers.

Based on the reviews, we are likely to accept this manuscript for publication, provided you satisfactorily address the following data and other policy-related requests.

IMPORTANT - please attend to the following:

a) Please address my Data Policy requests below; specifically, we need you to supply the numerical values underlying Figs 1BCDEFG, 2ABC, 3ABC, 4ABC, S3ABCD, S4, S5, S6, S7, S8, S9AB, either as a supplementary data file or as a permanent DOI’d deposition. I note that you already have an associated OSFdeposition (https://osf.io/aeg7p/) which seems to have a lot of data and code. Please could you confirm whether the data and code in this deposition are sufficient to recreate the Figures?

b) Please cite the location of the data clearly in all relevant main and supplementary Figure legends, e.g. “The data underlying this Figure can be found in S1 Data” or “The data underlying this Figure can be found in https://osf.io/aeg7p/"; also update your Data Availability Statement accordingly.

c) I note that you mention the editor and two of the reviewers ("We thank the editor and two anonymous reviewers") in the Acknowledgements. While we appreciate the sentiment, this is against PLOS policy, so please could you remove this?

d) Please make any custom code available, either as a supplementary file or as part of your data deposition.

As you address these items, please take this last chance to review your reference list to ensure that it is complete and correct. If you have cited papers that have been retracted, please include the rationale for doing so in the manuscript text, or remove these references and replace them with relevant current references. Any changes to the reference list should be mentioned in the cover letter that accompanies your revised manuscript.

In addition to these revisions, you may need to complete some formatting changes, which you will receive in a follow up email. A member of our team will be in touch with a set of requests shortly. If you do not receive a separate email within a few days, please assume that checks have been completed, and no additional changes are required.

We expect to receive your revised manuscript within two weeks.

To submit your revision, please go to https://www.editorialmanager.com/pbiology/ and log in as an Author. Click the link labelled 'Submissions Needing Revision' to find your submission record. Your revised submission must include the following:

- a cover letter that should detail your responses to any editorial requests, if applicable, and whether changes have been made to the reference list

- a Response to Reviewers file that provides a detailed response to the reviewers' comments (if applicable, if not applicable please do not delete your existing 'Response to Reviewers' file.)

- a track-changes file indicating any changes that you have made to the manuscript.

NOTE: If Supporting Information files are included with your article, note that these are not copyedited and will be published as they are submitted. Please ensure that these files are legible and of high quality (at least 300 dpi) in an easily accessible file format. For this reason, please be aware that any references listed in an SI file will not be indexed. For more information, see our Supporting Information guidelines:

https://journals.plos.org/plosbiology/s/supporting-information

*Published Peer Review History*

Please note that you may have the opportunity to make the peer review history publicly available. The record will include editor decision letters (with reviews) and your responses to reviewer comments. If eligible, we will contact you to opt in or out. Please see here for more details:

https://plos.org/published-peer-review-history/

*Press*

Should you, your institution's press office or the journal office choose to press release your paper, please ensure you have opted out of Early Article Posting on the submission form. We ask that you notify us as soon as possible if you or your institution is planning to press release the article.

*Protocols deposition*

To enhance the reproducibility of your results, we recommend that if applicable you deposit your laboratory protocols in protocols.io, where a protocol can be assigned its own identifier (DOI) such that it can be cited independently in the future. Additionally, PLOS ONE offers an option for publishing peer-reviewed Lab Protocol articles, which describe protocols hosted on protocols.io. Read more information on sharing protocols at https://plos.org/protocols?utm_medium=editorial-email&utm_source=authorletters&utm_campaign=protocols

Please do not hesitate to contact me should you have any questions.

Sincerely,

Roli Roberts

Roland Roberts, PhD

Senior Editor

rroberts@plos.org

PLOS Biology

------------------------------------------------------------------------

DATA POLICY:

You may be aware of the PLOS Data Policy, which requires that all data be made available without restriction: http://journals.plos.org/plosbiology/s/data-availability. For more information, please also see this editorial: http://dx.doi.org/10.1371/journal.pbio.1001797

Note that we do not require all raw data. Rather, we ask that all individual quantitative observations that underlie the data summarized in the figures and results of your paper be made available in one of the following forms:

1) Supplementary files (e.g., excel). Please ensure that all data files are uploaded as 'Supporting Information' and are invariably referred to (in the manuscript, figure legends, and the Description field when uploading your files) using the following format verbatim: S1 Data, S2 Data, etc. Multiple panels of a single or even several figures can be included as multiple sheets in one excel file that is saved using exactly the following convention: S1_Data.xlsx (using an underscore).

2) Deposition in a publicly available repository. Please also provide the accession code or a reviewer link so that we may view your data before publication.

Regardless of the method selected, please ensure that you provide the individual numerical values that underlie the summary data displayed in the following figure panels as they are essential for readers to assess your analysis and to reproduce it: Figs 1BCDEFG, 2ABC, 3ABC, 4ABC, S3ABCD, S4, S5, S6, S7, S8, S9AB. NOTE: the numerical data provided should include all replicates AND the way in which the plotted mean and errors were derived (it should not present only the mean/average values).

IMPORTANT: Please also ensure that figure legends in your manuscript include information on where the underlying data can be found, and ensure your supplemental data file/s has a legend.

Please ensure that your Data Statement in the submission system accurately describes where your data can be found.

------------------------------------------------------------------------

CODE POLICY

Per journal policy, if you have generated any custom code during the course of this investigation, please make it available without restrictions. Please ensure that the code is sufficiently well documented and reusable, and that your Data Statement in the Editorial Manager submission system accurately describes where your code can be found. More information on our Code Policy, what and how to share can be found here: https://journals.plos.org/plosbiology/s/code-availability

Please note that we cannot accept sole deposition of code in GitHub, as this could be changed after publication. However, you can archive this version of your publicly available GitHub code to Zenodo. Once you do this, it will generate a DOI number, which you will need to provide in the Data Accessibility Statement (you are welcome to also provide the GitHub access information). See the process for doing this here: https://docs.github.com/en/repositories/archiving-a-github-repository/referencing-and-citing-content

------------------------------------------------------------------------

DATA NOT SHOWN?

- Please note that per journal policy, we do not allow the mention of "data not shown", "personal communication", "manuscript in preparation" or other references to data that is not publicly available or contained within this manuscript. Please either remove mention of these data or provide figures presenting the results and the data underlying the figure(s).

------------------------------------------------------------------------

REVIEWERS' COMMENTS:

Reviewer #1:

I thank the authors for the careful consideration of my questions and suggestions. They have done an excellent job.

Reviewer #2:

The authors have adequately addressed my concerns. I congratulate the authors on this interesting work.

# Editor report: Decision Letter 3

**DOI:** 10.1371/journal.pbio.3004003.r005

**Authors:** Roland G Roberts

3 Sep 2026

Dear Dr Moran,

Thank you for the submission of your revised Research Article "Interindividual vocal tract diversity influences the phonetic diversification of spoken languages" for publication in PLOS Biology. On behalf of my colleagues and the Academic Editor, Catherine Hobaiter, I'm pleased to say that we can in principle accept your manuscript for publication, provided you address any remaining formatting and reporting issues. These will be detailed in an email you should receive within 2-3 business days from our colleagues in the journal operations team; no action is required from you until then. Please note that we will not be able to formally accept your manuscript and schedule it for publication until you have completed any requested changes.

Please take a minute to log into Editorial Manager at http://www.editorialmanager.com/pbiology/, click the "Update My Information" link at the top of the page, and update your user information to ensure an efficient production process.

PRESS: We frequently collaborate with press offices. If your institution or institutions have a press office, please notify them about your upcoming paper at this point, to enable them to help maximise its impact. If the press office is planning to promote your findings, we would be grateful if they could coordinate with biologypress@plos.org. If you have previously opted in to the early version process, we ask that you notify us immediately of any press plans so that we may opt out on your behalf.

We also ask that you take this opportunity to read our Embargo Policy regarding the discussion, promotion and media coverage of work that is yet to be published by PLOS. As your manuscript is not yet published, it is bound by the conditions of our Embargo Policy. Please be aware that this policy is in place both to ensure that any press coverage of your article is fully substantiated and to provide a direct link between such coverage and the published work. For full details of our Embargo Policy, please visit http://www.plos.org/about/media-inquiries/embargo-policy/.

Thank you again for choosing PLOS Biology for publication and supporting Open Access publishing. We look forward to publishing your study.

Sincerely,

Roli Roberts

Roland G Roberts, PhD, PhD

Senior Editor

PLOS Biology

rroberts@plos.org
